# Albuminuria in Lupus Nephritis: the hidden threat to cardiovascular health

**DOI:** 10.1093/rap/rkag032

**Published:** 2026-03-16

**Authors:** Julio Francisco Colina-García, Irene Martin Capón, María Galindo, Enrique Morales

**Affiliations:** Department of Nephrology, Hospital Universitario 12 de Octubre, Madrid, Spain; Department of Nephrology, Hospital Universitario 12 de Octubre, Madrid, Spain; Department of Rheumatology, Hospital Universitario 12 de Octubre, Madrid, Spain; Department of Medicine, Research Institute of Hospital Universitario 12 de Octubre (imas12), Madrid, Spain; Department of Medicine, Complutense University of Madrid, Madrid, Spain; Department of Nephrology, Hospital Universitario 12 de Octubre, Madrid, Spain; Department of Medicine, Research Institute of Hospital Universitario 12 de Octubre (imas12), Madrid, Spain; Department of Medicine, Complutense University of Madrid, Madrid, Spain; RICORS2040-Renal, ISCIII, Madrid, Spain

**Keywords:** albuminuria, LN, cardiovascular risk

## Abstract

**Objective:**

Systemic lupus erythematosus often leads to cardiovascular and renal complications, particularly in patients with lupus nephritis. In clinical practice, renal treatment response is commonly assessed using the Complete Renal Response criteria defined by the Consensus document of the Spanish Group for the Study of the Glomerular Diseases. These criteria, however, rely predominantly on proteinuria while albuminuria is often overlooked. The primary objective of our study was to assess the prevalence of pathological albuminuria in patients who met Complete Renal Response criteria. Secondary objectives included evaluating whether residual albuminuria identifies patients with different clinical outcomes.

**Methods:**

This single-centre, ambispective, observational study included 91 LN patients. Pathological albuminuria was defined as urinary albumin-creatinine ratio > 30 mg/g. Outcomes were compared between patients with and without pathological albuminuria despite achieving Complete Renal Response.

**Results:**

Among patients who met Complete Renal Response criteria, 80.5% had pathological albuminuria at the time of remission, and 55.8% continued to show it at the end of follow-up. Notably, patients with pathological albuminuria at the time of remission had significantly higher proteinuria levels both at disease onset and remission. Furthermore, persistent pathological albuminuria at follow-up was associated with more intensive antiproteinuric treatment. This therapeutic approach led to a significant reduction in both proteinuria and albuminuria titers.

**Conclusion:**

Pathological albuminuria remains common in lupus nephritis patients who meet Complete Renal Response criteria. Although cardiovascular events were infrequent during follow-up, the presence of persistent pathological albuminuria in patients meeting Complete Renal Response criteria underscores the need for close monitoring and therapeutic optimization, given its well-established association with adverse renal and cardiovascular outcomes in other glomerular diseases.

Key messages

- Pathological albuminuria frequently persists in LN patients despite achieving criteria for Complete Renal Response.
- Addressing pathological albuminuria might have long-term beneficial outcomes, as in other proteinuric chronic kidney diseases

## Introduction

SLE is a chronic autoimmune disorder associated with significant cardiovascular morbidity and mortality, particularly in cases with renal involvement [1–3]. Current clinical guidelines define complete renal remission (CRR) as proteinuria ≤ 0.5 g/day with an inactive urinary sediment (≤ 5 red blood cells per high power field [RBC/HPF]), serum albumin ≥ 3.5 g/dl and normal estimated glomerular filtration rate (eGFR) or ≤10% decline from baseline renal function at the time of the flare [4]. The treatment approach for patients with LN aims to preserve renal function by employing both immunosuppressive and non-immunosuppressive approaches, including the employment of antiproteinuric agents in order to optimize renal protection. Additionally, it aims to reduce overall cardiovascular risk through blood pressure and lipid control, as well as smoking cessation [5]. Despite these efforts, awareness of albuminuria as an independent cardiovascular risk factor in LN remains limited, with clinical attention primarily directed towards total proteinuria as a marker of renal status. However, emerging evidence suggests that moderate albuminuria (30–300 mg/day), even in the presence of preserved renal function, is associated with worsening of cardiorenal outcomes [6–8], as emphasized in the KDIGO guidelines [9]. This fact underscores the need to reconsider standard criteria for renal response to improve prognostic accuracy and therapeutic strategies for SLE patients.

Renin-angiotensin-aldosterone system inhibitors (RAASi), including angiotensin-converting enzyme inhibitors (ACEi) or angiotensin-II receptor blockers (ARBs), represent the most widely used non-immunosuppressive treatment for reducing proteinuria in glomerular diseases. In addition, sodium-glucose cotransporter-2 inhibitors (SGLT2i) have emerged as a potential adjunctive therapy for this purpose [10, 11].

To date, the proportion of patients with LN meeting the criteria for CRR who exhibit pathological albuminuria remains unknown. In contrast, in routine clinical practice, the assessment of albuminuria alongside total proteinuria is becoming increasingly common due to its impact on cardiovascular risk in patients with chronic conditions.

In this study, we aimed to assess the prevalence of pathological albuminuria in LN who meet criteria for CRR, and whether there are clinical, histological or prognostic differences in patients with pathological albuminuria compared with those with normal albuminuria.

## Methods

### Study population and design

We conducted a single-centre, ambispective, observational study including patients followed at our interdisciplinary LN clinic. All patients presented biopsy-proven LN that was classified following the ISN/RPS criteria (International Society of Nephrology/Renal Pathology Society) [1].

This study was conducted in compliance with the Organic Law 3/2018 on the Protection of Personal Data and Guarantee of Digital Rights, and with Regulation (EU) 2016/679 of the European Parliament and of the Council of 27 April 2016 (General Data Protection Regulation). In accordance with Spanish regulations (Real Decreto 957/2020 and applicable data protection laws), formal ethics committee approval and written informed consent were deemed unnecessary for this retrospective observational study based on anonymized existing data.

### Clinical and laboratory data

Data from the included patients at LN onset and at the end of follow-up were retrieved from their medical records. Collected clinical data included age, gender, race/ethnicity, histological class of LN and the concomitant presence of antiphospholipid antibodies (APAs) or APS. Laboratory data included serum creatinine (sCr) levels, eGFR, ANA titer, anti-double-strain-DNA antibodies (anti-dsDNA) titer, complement C3 and C4 levels, the number of RBC/HPF in urinary sediment, albuminuria expressed as urinary albumin-creatinine ratio (uACR, measured in mg/g) and proteinuria expressed as urinary protein-creatinine ratio (uPCR, measured in g/g). The presence of other cardiovascular risk factors, such as high blood pressure (HBP), type 2 diabetes mellitus (T2DM), dyslipidaemia (DL), BMI and smoking habit, was also documented.

### Definitions

Complete Renal Response was defined according to the recommendations of the Spanish Group for the Study of Glomerular Diseases for the Diagnosis and Treatment of LN [2], as proteinuria ≤ 0.5 g/day, inactive urinary sediment (≤ 5 red blood cells per high-power field [RBC/HPF]), serum albumin ≥ 3.5 g/dl and either a normal eGFR or ≤10% lower than that existing before the outbreak.

Pathological albuminuria was defined as albuminuria >30 mg/g and was categorized into three grades based on severity: A1 (normal to mild), with albuminuria <30 mg/g; A2 (moderate), with albuminuria between 30 and 300 mg/g; and A3 (severe), with albuminuria >300 mg/g.

Cardiovascular events were defined as major adverse cardiovascular events (MACEs), including myocardial infarction and stroke.

All available data were included up to the last follow-up visit, and losses to follow-up or patient withdrawals were recorded; no imputation was performed for missing outcomes.

### Outcomes

Our primary outcome was to assess the prevalence of pathological albuminuria among patients with LN after achieving CRR.

As secondary objectives, we determined:

- Whether differences existed between patients with pathological albuminuria and those without albuminuria in relation to the following parameters:
   ○ Renal parameters: sCr, eGFR and total proteinuria.
   ○ Immunological parameters: C3 and C4 titer (normal values in our institution for C3 and C4 are 83–171 and 14–38 mg/dl, respectively), ANA and anti-dsDNA status, CRP, SLEDAI 2000 (SLEDAI-2K) [3], SLEDAS [4].
   ○ Antiproteinuric agent(s) employed.
   ○ Immunosuppressive treatment used.
   ○ Cardiovascular parameters: cardiovascular events, fasting glucose, cholesterol, blood pressure.
- The proportion of patients in whom antiproteinuric treatment was intensified.
- The percentage reduction in proteinuria following treatment modification.
- Whether there were differences in final albuminuria following the intensification of antiproteinuric treatment.

### Statistical analysis

A descriptive analysis was first performed. Categorical data were expressed as counts and percentages. Continuous data were expressed as mean and SD or, when non-normally distributed, as median and interquartile range (IQR). Normality was assessed by using the Kolmogorov–Smirnov test.

Subsequently, a comparative analysis was performed between patients with CRR and either pathological or normal albuminuria at remission. Comparisons were performed with the t-test for normally distributed continuous variables and Mann–Whitney U test for non-normally distributed continuous variables. Statistical analyses were conducted using IBM SPSS Version 25.0.

## Results

### Baseline clinical, immunological and histological characteristics at disease onset

A total of 91 patients were included in our study, of whom 77 (84.6%) achieved CRR. Baseline characteristics of patients that met the criteria for CRR are summarized in Table 1, whereas Supplementary Table S1, available at *Rheumatology Advances in Practice* Online, displays the baseline characteristics at LN onset among the overall cohort.

**Table 1:** Baseline characteristics at onset among patients with LN and complete remission.

Patients, *N*	77
**Clinical characteristics**	
Female/male, *N* (%)	68/9 (88.3/11.7)
Race/ethnicity, *N* (%)	
Caucasian	35 (45.4)
Hispanic	31 (40.3)
Asian	7 (9.1)
African	4 (5.2)
Age, years (M ± SD)	33.47 ± 14.16
Time to remission, months (median, IQR)	28 (12–98)
Time of follow-up, months (median, IQR)	78 (38.5–201)
Concomitant APS or APA, *N* (%)	23 (29.9)
HBP, *N* (%)	34 (44.2)
Systolic blood pressure, mmHg (M ± SD)	127 ± 15.76
Diastolic blood pressure, mmHg (M ± SD)	81.38 ± 10.11
T2DM, *N* (%)	1 (1.3)
DL, *N* (%)	31 (40.3)
Active smoker, *N* (%)	9 (11.7)
**Histological characteristics**	
ISN/RPS histological classification, *N* (%)	
Class II	3 (3.8)
Class III	13 (16.9)
Class IV	29 (37.7)
Class V	15 (19.5)
Class III + V	8 (10.4)
Class IV + V	9 (11.7)
Activity Index (median, IQR)	4 (0–8)
Chronicity Index (median, IQR)	1 (0–2)
**Biochemical characteristics**	
sCr titer, mg/dl, (M ± SD)	0.97 ± 0.77
eGFR ml/min, (M ± SD)	81.94 ± 15.82
uPCR, g/g (median, IQR)	1.77 (0.62–4.93)
uACR, mg/g (median, IQR)	931.15 (402.74–2944.61)
Fasting blood glucose (M ± SD)	86.41 ± 7.67
Total cholesterol titer, mg/dl (M ± SD)	182.61 ± 35.20
LDL-cholesterol titer, mg/dl (M ± SD)	102 ± 27.56
Triglycerides titer, mg/dl (M ± SD)	92.48 ± 36.84
**Immunological features**	
C3 titer, mg/dl (median, IQR)	59.55 (44.62–82.82)
C4 titer, mg/dl (median, IQR)	10.60 (5.39–18.25)
ANA status positive, *N* (%)	45 (58.4)
Anti-dsDNA status positive, *N* (%)	32 (41.6)
SLEDAI-2K (median, IQR)	14 (11–20)
SLE-DAS (median, IQR)	20.10 (14.18–33.73)

HBP: high blood pressure; T2DM: type 2 diabetes mellitus; DL: dyslipidaemia; ISN/RPS: International Society of Nephrology/Renal Pathology Society; sCr: serum creatinine; eGFR: estimated glomerular filtration rate; uPCR: urinary protein-creatinine ratio; uACR: urinary albumin-creatinine ratio; SLEDAI-2K: SLEDAI 2000; M ± SD: mean ± SD; IQR: interquartile range.

Most participants were female (88.3% vs 11.7%). Regarding racial and ethnic distribution, 45.4% of the patients were Caucasian, followed by 40.3% Hispanic, 9.1% Asian and 5.2% African. The mean age at LN onset was 33.47 ± 14.16 years, while the mean age at the end of follow-up was 45.71 ± 14.05 years. Regarding comorbidities, HBP was present in 44.2% of the cohort, DL in 40.3% and T2DM in 1.3%. Additionally, 11.7% of patients were active smokers. The presence of concomitant APS or APAs was found in 29.9% of the cohort.

Based on the histological classification established by the ISN/RPS, Class IV LN was the most prevalent histological subtype (37.7% of patients), followed by Class V (19.5%) and Class III (16.9%). Mixed forms (III+V or IV+V) were present in 22.1% of participants. Median Activity Index was 4 (0–8) and median Chronicity Index was 1 (0–2).

Mean Cr and eGFR at disease onset were 0.97 ± 0.77 mg/dl and 81.94 ± 15.82 ml/min, respectively. Median uPCR was 1.77 [0.62–4.93] g/g and median uACR was 931 [402.74–2944.61] mg/g. At the time of LN diagnosis, 58.4% of patients were ANA positive, and 41.6% were anti-dsDNA positive. C3 and C4 titer at diagnosis showed a median of 59.55 [44.62–82.82] and 10.60 [5.39–18.25] mg/dl, respectively. Time to remission was 28 [12–98] months, and time of follow-up was 78 [38.5–201] months.

### Comparison analysis between patients meeting criteria for CRR and normal or pathological albuminuria

**Figure 1:**
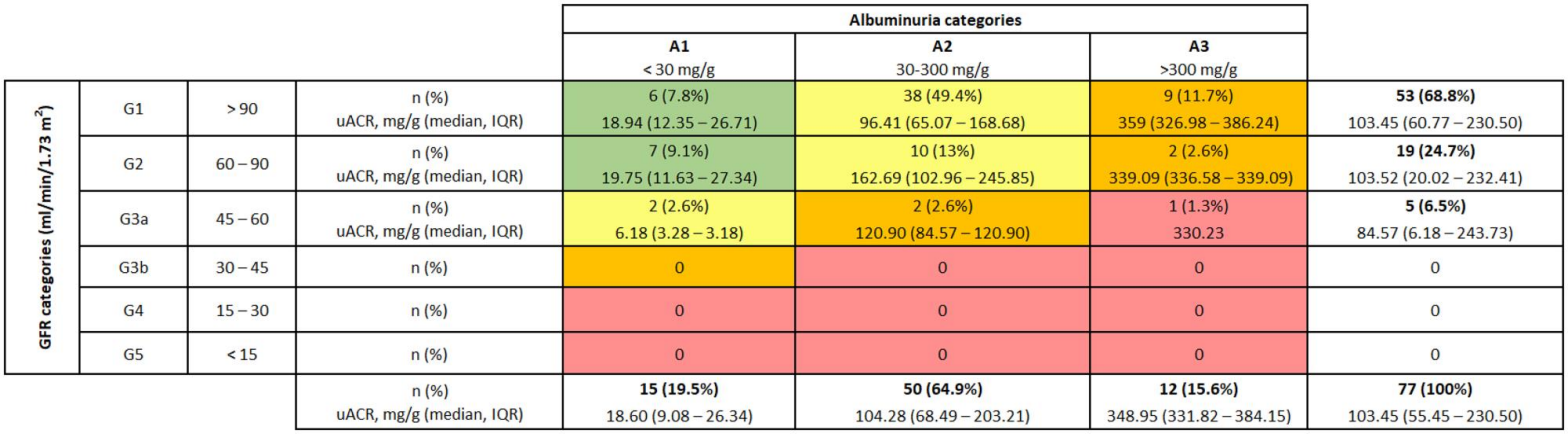
Cohort distribution based on their eGFR and albuminuria classification, as defined by the KDIGO guidelines, among patients that achieved CRR. GFR: glomerular filtration rate; uACR: urinary albumin-creatinine ratio; IQR: interquartile range

Among the 77 patients with renal CRR, 62 (80.5%) exhibited persistent pathological albuminuria at the time of remission. Figure 1 illustrates the distribution of participants based on their eGFR and albuminuria classification, as defined by the KDIGO guidelines [9]. When classified by severity, 19.5% of patients presented A1 albuminuria, 64.9% had A2 albuminuria and 15.6% had A3 albuminuria.

At baseline, patients with CRR and either normal or pathological albuminuria exhibited no significant differences in clinical or histological characteristics. However, at the time of remission, those with pathological albuminuria had significantly higher titer of albuminuria and proteinuria at disease onset compared with patients with normal albuminuria, as reflected by median uACR values of 1230.54 [531.87–3174.47] vs 468.91 [178.69–728.56] mg/g (*P* < 0.001), and median uPCR values of 2.29 [1.03–5.05] vs. 1.08 [0.37–1.72] g/g (*P* < 0.001). These data are detailed in Table 2.

**Table 2:** Comparison of baseline characteristics between patients with complete remission and pathological albuminuria and those without pathological albuminuria.

	Patients meeting criteria for CR		
	Normal albuminuria (*N *= 15)	Pathological albuminuria (*N* = 62)	*P*
**Clinical characteristics**			
Female/male, *N* (%)	12/3 (80/20)	56/6 (90.3/9.7)	0.264
Age at LN onset, years (M ± SD)	30.73 ± 11.67	34.13 ± 14.71	0.408
Race/ethnicity, *N* (%)			0.317
Caucasian	6 (40)	29 (46.8)	
Hispanic	6 (40)	25 (40.3)	
Asian	3 (20)	4 (6.5)	
African	0 (0)	4 (6.5)	
Concurrent APS or APA, *N* (%)	8 (53.3)	15 (24.2)	**0.027**
LN flares, *N* (%)	2 (13.3)	10 (16.7)	0.753
Time to remission, months (median, IQR)	22.5 (12–150)	33 (9–95)	0.408
**Histological characteristics**			
ISN/RPS histological classification, *N* (%)			0.404
Class II	1 (6.7)	2 (3.2)	
Class III	2 (13.3)	11 (17.7)	
Class IV	7 (46.7)	22 (35.5)	
Class V	2 (13.3)	13 (21)	
Class III + V	3 (20)	5 (8.1)	
Class IV + V	0 (0)	9 (14.5)	
Activity Index (median, IQR)	7 (0.50–8)	4 (0–8)	0.444
Chronicity Index (median, IQR)	0 (0–2)	1 (0–2)	0.601
**Biochemical characteristics**			
sCr titer at LN onset, mg/dl (M ± SD)	1.25 ± 0.97	0.89 ± 0.43	0.278
eGFR at LN onset, ml/min (M ± SD)	84.40 ± 7.70	81.53 ± 16.85	0.713
uPCR at LN onset, g/g (median, IQR)	1.08 (0.37–1.72)	2.29 (1.03–5.05)	**<0.001**
uACR at LN onset, mg/g (median, IQR)	468.91 (178.69–728.56)	1230.54 (531.87–3174.47)	**<0.001**
Albuminuria classification at LN onset, *N* (%)			0.153
A1: < 30 mg/g	0 (0)	0 (0)	
A2: 30–300 mg/g	3 (42.9)	7 (18.4)	
A3: > 300 mg/g	4 (57.1)	31 (81.6)	
**Immunological features**			
C3 titer, mg/dl (median, IQR)	61.5 (47.1–74.05)	59.20 (43.60–91.50)	0.729
C4 titer, mg/dl (median, IQR)	14.55 (9.26–24.83)	8.84 (4.77–18.10)	0.096
ANA status positive, *N* (%)	10 (66.7)	35 (56.5)	0.471
Anti-dsDNA status positive, *N* (%)	7 (46.7)	25 (40.3)	0.655
SLEDAI-2K (median, IQR)	14 (10.25–19.25)	14 (11–21)	0.646
SLE-DAS (median, IQR)	19.36 (8.75–20.03)	23.10 (15.93–36.89)	**0.008**

**Bold text** in the tables indicates statistically significant results (*P* < 0.05).

ISN/RPS: International Society of Nephrology/Renal Pathology Society; sCr: serum creatinine; eGFR: estimated glomerular filtration rate; uPCR: urinary protein-creatinine ratio; uACR: urinary albumin-creatinine ratio; SLEDAI-2K: SLEDAI 2000; M ± SD: mean ± SD; IQR: interquartile range.

At the time of achieving CRR, both uPCR and uACR titer were significantly higher in the pathological albuminuria group. Although median uPCR values remained below 0.5 g/g in both groups, patients with persistent pathological albuminuria had significantly higher uPCR titer [0.36 (0.22–0.44) vs 0.10 (0.09–0.23) g/g; *P* < 0.001]. No statistically significant differences were observed in other renal, immunological, cardiovascular or therapeutic features. Notably, only one patient in the pathological albuminuria group experienced a major cardiovascular event (ischemic stroke). The full comparative data are provided in Table 3.

**Table 3:** Comparison of outcomes at the time of renal complete remission between patients with pathological albuminuria and those without pathological albuminuria.

	Patients meeting criteria for CR		
	Normal albuminuria (*N* = 15)	Pathological albuminuria (*N* = 62)	*P*
**Renal outcomes**			
sCr titer, mg/dl (M ± SD)	0.81 ± 0.23	0.74 ± 0.19	0.273
eGFR, ml/min (M ± SD)	88.09 ± 21.05	84.46 ± 11.16	0.430
uPCR, g/g (median, IQR)	0.10 (0.09–0.23)	0.36 (0.22–0.44)	**<0.001**
uACR, mg/g (median, IQR)	18.60 (9.08–26.34)	126.17 (77.30–269.16)	**<0.001**
Albuminuria severity, *N* (%)			**<0.001**
A1: < 30 mg/g	15 (100)	0 (0)	
A2: 30–300 mg/g	0 (0)	50 (80.6)	
A3: > 300 mg/g	0 (0)	12 (19.4)	
Number of antiproteinuric agents prescribed (median, IQR)	1 (0–1)	1 (0–2)	0.155
None, *N* (%)	6 (42.9)	16 (27.1)	
1 agent, *N* (%)	6 (42.9)	26 (44.1)	
2 agents, *N* (%)	2 (14.3)	14 (23.7)	
3 agents, *N* (%)	0 (0)	3 (5.1)	
Class of antiproteinuric agent administered, *N* (%)			
ACEi/ARBs	8 (53.3)	40 (64.5)	0.724
SGLT2i	0 (0)	5 (8.1)	0.512
Thiazides	1 (7.1)	4 (6.8)	0.961
MRAs	1 (7.1)	11 (18.6)	0.297
**Immunological outcomes**			
Low C3, *N* (%)	8 (53.3)	30 (48.4)	0.731
Low C4, *N* (%)	6 (40)	22 (35.5)	0.744
C3 titer, mg/dl (median, IQR)	81.50 (71.43–98.42)	81 (61.80–99)	0.587
C4 titer, mg/dl (median, IQR)	15.90 (12.72–19.83)	14.70 (11–20.40)	0.484
ANA status positive, *N* (%)	11 (73.3)	35 (56.5)	0.232
Anti-dsDNA status positive, *N* (%)	6 (40)	14 (22.6)	0.167
SLEDAI-2K (median, IQR)	0 (0–1)	0 (0–2)	0.506
SLE-DAS (M ± SD)	0.37 (0.37–1.12)	0.37 (0.37–1.12)	0.451
**Cardiovascular outcomes**			
Cardiovascular events, *N* (%)	0 (0)	1 (1.6)	0.621
Class of cardiovascular event		Stroke	

**Bold text** in the tables indicates statistically significant results (*P* < 0.05).

sCr: serum creatinine; eGFR: estimated glomerular filtration rate; uPCR: urinary protein-creatinine ratio; uACR: urinary albumin-creatinine ratio; ACEi/ARBs: angiotensin-converting enzyme inhibitors/angiotensin II receptor blockers; SGLT2i: sodium-glucose cotransporter-2 inhibitors; MRAs: mineralocorticoid receptor antagonists; SLEDAI-2K: SLEDAI 2000; M ± SD: mean ± SD; IQR: interquartile range.

At the end of follow-up, among the 77 patients who achieved renal remission, 34 (44.2%) exhibited normal albuminuria, while 43 (55.8%) persisted with pathological albuminuria. No statistically significant differences were observed between both groups regarding demographic characteristics, clinical presentation, histological class at diagnosis or immunological parameters. A summary of the group comparison at the end of follow-up is presented in Table 4.

**Table 4:** Comparison of outcomes at the end of follow-up between patients meeting criteria for CRR exhibiting persistent pathological albuminuria and those without pathological albuminuria.

	Patients meeting criteria for CR		
	Normal albuminuria (*N* = 34)	Pathological albuminuria (*N* = 43)	*P*
**Clinical baseline characteristics**			
Female/male, *N* (%)	32/2 (94.1/5.9)	36/7 (83.7/16.3)	0.305
Age at LN onset, years (M ± SD)	31.94 ± 14.28	34.67 ± 14.24	0.404
Race/ethnicity, *N* (%)			0.374
Caucasian	16 (47.1)	19 (44.2)	
Hispanic	12 (35.3)	19 (44.2)	
Asian	5 (14.7)	2 (4.6)	
African	1 (2.9)	3 (7)	
Concurrent APS or APA, *N* (%)	11 (32.4)	12 (27.9)	0.672
BMI at the end of follow-up, kg/m^2^ (median, IQR)	23.50 (21.10–29)	25.50 (22.23–28.63)	0.263
LN flares, *N* (%)	4 (12.5)	8 (18.6)	0.476
uPCR at LN onset, g/g (median, IQR)	1.74 (0.55–4.16)	1.81 (0.96–5.04)	0.434
uACR at LN onset, mg/g (median, IQR)	708.21 (272.64–2204.67)	1064.01 (540.20–3115.76)	0.451
Follow-up time, months (median, IQR)	115.50 (46–212.25)	73 (33–181)	0.244
Time to remission, months (median, IQR)	29 (12–95)	27.50 (8.75–105.25)	0.445
**Histological baseline features**			
ISN/RPS histological classification, *N* (%)			0.253
Class II	2 (5.9)	1 (2.3)	
Class III	7 (20.6)	6 (14)	
Class IV	15 (44.1)	14 (32.6)	
Class V	5 (14.7)	10 (23.3)	
Class III + V	4 (11.8)	4 (9.3)	
Class IV + V	1 (2.9)	8 (18.6)	
Activity Index (median, IQR)	3.50 (0–8)	4 (0.25–8)	0.423
Chronicity Index (median, IQR)	0 (0–2)	1 (0–2.75)	0.269
**Renal outcomes**			
sCr titer, mg/dl (M ± SD)	0.87 ± 0.47	0.92 ± 0.43	0.620
eGFR, ml/min (M ± SD)	78.91 ± 19.30	76.35 ± 20.13	0.574
uPCR at the time of renal remission, g/g (median, IQR)	0.25 (0.10–0.39)	0.36 (0.21–0.45)	**0.007**
uACR at the time of renal remission, mg/g (median, IQR)	53.23 (19.11–168.68)	132.40 (78.64–294.16)	**0.006**
uPCR at the end of follow-up g/g (median, IQR)	0.09 (0.08–0.13)	0.21 (0.16–0.33)	**<0.001**
uACR at the end of follow-up, mg/g (median, IQR)	10.55 (3.71–20.03)	77.73 (56.17–147.38)	**<0.001**
Albuminuria severity, *N* (%)			**<0.001**
A1: <30 mg/g	34 (100)	0 (0)	
A2: 30–300 mg/g	0 (0)	40 (93)	
A3: >300 mg/g	0 (0)	3 (7)	
Number of antiproteinuric agents prescribed (median, IQR)	1 (0–1)	1 (1–3)	**0.002**
None, *N* (%)	10 (29.5)	6 (14)	
1 agent, *N* (%)	17 (50)	17 (39.5)	
2 agents, *N* (%)	6 (17.6)	9 (20.9)	
3 agents, *N* (%)	1 (2.9)	10 (23.3)	
4 agents, *N* (%)	0 (0)	1 (2.3)	
Class of antiproteinuric agent administered, *N* (%)			
ACEi/ARBs	24 (70.6)	36 (83.7)	0.168
SGLT2i	1 (2.9)	13 (30.2)	**0.002**
Thiazides	3 (8.8)	7 (16.3)	0.334
MRAs	3 (8.8)	12 (27.9)	**0.036**
**Immunological outcomes**			
Low C3, *N* (%)	16 (47.1)	24 (55.8)	0.445
Low C4, *N* (%)	14 (41.2)	13 (30.2)	0.318
C3 titer, mg/dl (median, IQR)	84.95 (63.30–98.25)	81.70 (73.90–98)	0.758
C4 titer, mg/dl (median, IQR)	16.95 (12.20–23.63)	16.30 (13.70–23.90)	0.558
ANA status positive, *N* (%)	6 (17.6)	6 (14)	0.657
Anti-dsDNA status positive, *N* (%)	4 (11.8)	4 (9.3)	0.725
SLEDAI-2K (median, IQR)	0 (0–2)	0 (0–2)	0.291
SLE-DAS (M ± SD)	1.01 ± 1.51	1.02 ± 1.18	0.982
**Cardiovascular outcomes**			
Cardiovascular events, *N* (%)	0 (0)	1 (2.3)	0.371
Class of cardiovascular event		Stroke	
Fasting glucose, mg/dl (M ± SD)	85.74 ± 6.84	87.09 ± 8.53	0.558

**Bold text** in the tables indicates statistically significant results (*P* < 0.05).

uPCR: urinary protein-creatinine ratio; uACR: urinary albumin-creatinine ratio; sCr: serum creatinine; eGFR: estimated filtrate glomerular rate; ACEi/ARBs: angiotensin-converting enzyme inhibitors/angiotensin II receptor blockers; SGLT2i: sodium-glucose cotransporter-2 inhibitors; MRAs: mineralocorticoid receptor antagonists; SLEDAI-2K: SLEDAI 2000; M ± SD: mean ± SD; IQR: interquartile range.

Renal function at follow-up was similar between groups in terms of sCr and eGFR. However, patients with pathological albuminuria exhibited significantly higher uPCR at remission [0.25 (0.10–0.39) vs 0.36 (0.21–0.45) g/g; *P* = 0.007] and uACR [53.23 (19.11–168.68) vs 132.40 (78.64–294.16) mg/g; *P* = 0.006]. This trend persisted at the end of follow-up [median uPCR 0.09 (0.08–0.13) vs 0.21 (0.16–0.33) g/g, *P* < 0.001; median uACR 10.55 (3.71–20.03) vs 77.73 (56.17–147.38) mg/g, *P* < 0.001].

Among the participants who met criteria for CRR and persisted with pathological albuminuria at the end of follow-up, antiproteinuric therapy was intensified in 30 cases (48.6%). This treatment approach concluded in a significant reduction of both proteinuria and albuminuria, as reflected by the median uPCR and uACR values of 0.16 [0.10–0.28] vs 0.29 [0.18–0.42] g/g, *P* < 0.001; and 43.90 mg/g [11.28–99.47] vs 103.45 [55.45–230.50] g/g, *P* < 0.001, respectively. The most commonly used antiproteinuric agents at the end of follow-up were ACEi/ARBs (77.9%) and mineralocorticoid receptor antagonists (MRAs) (19.5%), followed by SGLT2i (18.2%) and thiazides (13%). Additionally, most participants were undergoing treatment with hydroxychloroquine (70.9%), while statins and ASA were administered in 44.2% and 31.2% of participants, respectively. The patients who were not receiving active hydroxychloroquine treatment had discontinued it due to documented drug toxicity. Regarding immunosuppressive treatment at the end of follow-up, MMF monotherapy was the most prevalent regimen, present in 24.7% of patients, followed by combination of glucocorticoid (GC) + MMF regimen (11.7%) and GC + MMF + calcineurin inhibitors (CNi) + belimumab (6.5%). Data regarding this subject are displayed in Supplementary Table S2, available at *Rheumatology Advances in Practice* Online.

Additionally, a significantly higher proportion of patients undergoing treatment with MRAs and SGLT2i was observed among those with persistent albuminuria, at the end of follow-up (*P* = 0.036 and *P* = 0.002, respectively). Moreover, the group with pathological albuminuria underwent a more intensive antiproteinuric regimen, as reflected by the greater number of agents administered [median 1 (0–1) vs 1 (1–3) agents; *P* = 0.001].

Other clinical, immunological and biochemical characteristics at the end of follow-up are reflected in Supplementary Table S3, available at *Rheumatology Advances in Practice* Online.

### Comparison analysis between patients meeting criteria for renal response with pathological albuminuria, at the moment of renal remission and at the end of follow-up

A significant reduction in the proportion of patients with persistent pathological albuminuria despite normal proteinuria was achieved at the end of follow-up (80.5% vs 55.8%, *P* = 0.004). This improvement occurred in the setting of an intensified antiproteinuric therapeutic approach, as evidenced by a higher median number of antiproteinuric agents used at follow-up compared with at the time of renal remission [1 (1–3) vs 1 (0–2), *P* = 0.001]. Notably, the proportion of patients receiving SGLT2i significantly increased during this period (8.1% vs 30.2%, *P* = 0.004). This therapeutic approach was associated with a significant reduction of both proteinuria and albuminuria values at the end of follow-up, with a median uPCR of 0.36 [0.22–0.44] vs 0.21 [0.16–0.33] g/g, *P* = 0.004; and a median uACR of 126.17 [77.30–269.16] vs 77.73 [56.17–147.38] mg/g, *P* = 0.002. In addition, the proportion of patients with A3 albuminuria significantly declined from 19.4% to 7% (*P* = 0.016).

Regarding immunological outcomes, the reduction in persistent pathological albuminuria was also accompanied by a significant increase in C4 titers (*P* = 0.022) and a lower proportion of patients testing positive for ANA (56.5% vs 14%, *P* < 0.001) and anti-dsDNA antibodies (22.6% vs 9.3%, *P* = 0.004; respectively). Full data on these outcomes are detailed in Table 5.

**Table 5:** Characteristics of patients with CRR and pathological albuminuria before and after antiproteinuric treatment intensification.

	Patients meeting criteria for CR with pathological albuminuria		
	At the time of remission	At the end of follow-up	*P*
Patients, *N* (%)	62 (80.5)	43 (55.8)	**0.004**
Female/male, *N* (%)	56/6 (90.3/9.7)	36/7 (83.7/16.3)	0.159
Age at LN onset, years (M ± SD)	34.13 ± 14.71	34.67 ± 14.24	0.147
Concurrent APS, *N* (%)	15 (24.2)	12 (27.9)	0.672
sCr titer, mg/dl (M ± SD)	0.74 ± 0.19	0.92 ± 0.43	0.765
eGFR, ml/min (M ± SD)	84.46 ± 11.16	76.35 ± 20.13	0.470
uPCR, g/g (median, IQR)	0.36 (0.22–0.44)	0.21 (0.16–0.33)	**0.004**
uACR mg/g (median, IQR)	126.17 (77.30–269.16)	77.73 (56.17–147.38)	**0.002**
Albuminuria severity, *N* (%)			**0.016**
A2: 30–300 mg/g	50 (80.6)	40 (93)	
A3: >300 mg/g	12 (19.4)	3 (7)	
Number of antiproteinuric agents prescribed (median, IQR)	1 (0–2)	1 (1–3)	**0.001**
None, *N* (%)	19 (30.7)	6 (14)	
1 antiproteinuric, *N* (%)	26 (41.9)	17 (39.5)	
2 antiproteinurics, *N* (%)	14 (22.6)	9 (20.9)	
3 antiproteinurics, *N* (%)	3 (4.8)	10 (23.3)	
4 antiproteinurics, *N* (%)	0 (0)	1 (2.3)	
Class of antiproteinuric agent administered, *N* (%)			
ACEi/ARBs	40 (64.5)	36 (83.7)	0.077
SGLT2i	5 (8.1)	13 (30.2)	**0.004**
Thiazides	4 (6.8)	7 (16.3)	0.227
MRAs	11 (18.6)	12 (27.9)	0.754
Low C3, *N* (%)	30 (48.4)	24 (55.8)	0.845
Low C4, *N* (%)	22 (35.5)	13 (30.2)	1
C3 titer, mg/dl (median, IQR)	81 (61.80–99)	81.70 (73.90–98)	0.344
C4 titer, mg/dl (median, IQR)	14.70 (11–20.40)	16.30 (13.70–23.90)	**0.022**
ANA status positive, *N* (%)	35 (56.5)	6 (14)	**<0.001**
Anti-dsDNA status positive, *N* (%)	14 (22.6)	4 (9.3)	**0.004**
SLEDAI-2K (median, IQR)	0 (0–2)	0 (0–2)	0.647
SLE-DAS (median, IQR)	0.37 (0.37–1.12)	0.37 (0.37–1.12)	0.447

**Bold text** in the tables indicates statistically significant results (*P* < 0.05).

uPCR: urinary protein-creatinine ratio; uACR: urinary albumin-creatinine ratio; ACEi/ARBs: angiotensin-converting enzyme inhibitors/angiotensin II receptor blockers; SGLT2i: sodium-glucose cotransporter-2 inhibitors; MRAs: mineralocorticoid receptor antagonists; SLEDAI-2K: SLEDAI 2000; M ± SD: mean ± SD; IQR: interquartile range.

A supplementary analysis compared patients who experienced normalization of albuminuria during follow-up with those who did not. No statistically significant differences were observed across the evaluated parameters.

A comprehensive analysis of classic cardiovascular risk factors revealed no significant differences in the prevalence of HBP, T2DM, DL or smoking habits between patients who fulfilled the criteria for CRR with pathological albuminuria and those with normal albuminuria. Moreover, linear regression analysis did not identify any outcome associated with the persistence of pathological albuminuria in the presence of normal proteinuria.

## Discussion

The primary finding of our analysis was that more than half of the participants who met the criteria for CRR continued to exhibit pathological albuminuria, both at the time of achieving remission and at the end of follow-up.

In our cohort, 80.5% of patients that met criteria for CRR exhibited pathological albuminuria at the time of achieving remission, whereas 55.8% continued to present pathological albuminuria at the end of follow-up. Specifically, 93% of patients presented with A2 albuminuria, and 7% with A3 albuminuria at the end of follow-up. Importantly, sCr titer and eGFR, whether at disease onset or at the end of follow-up, were not associated with the presence of pathological albuminuria at either time point. In contrast, patients with pathological albuminuria at the time of achieving remission had significantly higher overall proteinuria both at disease onset and at the time of remission (still within the remission range of <0.5 g/g), compared with those without pathological albuminuria. Similarly, the persistence of pathological albuminuria at the end of follow-up was associated with both higher global proteinuria and albuminuria at the time of remission.

The clinical implications of these findings are significant. Albuminuria is a relevant biomarker of generalized endothelial damage, particularly within the glomerular capillary wall, as well as increased vascular permeability resulting from inflammatory processes of diverse origins in the vasculature [5]. Given this association, it is not surprising that albuminuria has been established as an independent risk factor for both cardiovascular disease (CVD) and chronic kidney disease (CKD) by multiple meta-analysis [5–9]. This evidence has led the KDIGO guidelines to recognize albuminuria as a more sensitive marker of kidney injury than proteinuria [10], especially in the context of LN and other glomerular diseases, where inflammatory processes can lead to subtle yet persistent renal injury. Therefore, our results reflect that a substantial proportion of our cohort remains at an elevated risk for adverse renal and cardiovascular outcomes despite meeting renal remission criteria.

However, despite its well-established prognostic value, albuminuria remains underrepresented in current LN remission criteria, which continue to primarily rely on proteinuria, haematuria and eGFR—even though the absence of proteinuria does not necessarily exclude ongoing histological activity [11]. In this context, recent publications have raised concerns regarding certain aspects of the latest EULAR/ERA-EDTA recommendations for LN [12, 13]. These guidelines do not establish proteinuria targets beyond the 12-month mark, nor do they address albuminuria thresholds, thereby creating uncertainty in long-term renal management and constituting a critical gap in patient care. In contrast, in other CKD populations—particularly diabetic nephropathy—albuminuria is a well-established therapeutic target. By analogy, LN patients with persistent albuminuria should be considered at high risk and managed with a nephroprotective strategy similar to that used in diabetic or hypertensive CKD. Given these limitations, it is crucial for nephrologists and rheumatologists to critically evaluate these criteria and refine them in light of emerging evidence to ensure they remain clinically relevant and reflective of real-world patient evolution.

Notably, in our cohort, normalization of albuminuria among patients who met the criteria for CRR was associated with a significant increase in C4 titers and a reduction in anti-dsDNA antibody titers. Although some patients also showed ANA negativization, this finding is not typically considered a key marker of disease activity or treatment response in LN and should be interpreted cautiously.

In addition, the persistence of pathological albuminuria at the end of follow-up was associated with the implementation of a more intensive antiproteinuric regimen, which included a significantly higher use of SGLT2 inhibitors. Importantly, this therapeutic approach was associated with several statistically significant benefits, including a reduction in the proportion of patients with pathological albuminuria among those meeting renal response criteria, as well as decreased median titer of both uPCR and uACR. Furthermore, the proportion of patients with A3-grade albuminuria was notably lower under this intensified treatment approach.

Complementary to the immunosuppressive approach for the management of LN, classical non-immunosuppressive drugs—such as RAASi and diuretics—are widely employed among LN patients due to their well-established cardio-renal benefits and antiproteinuric properties [14]. However, increasing attention is being given to emerging non-immunosuppressive agents that also exhibit both cardio and nephroprotective properties. In this context, there is extensive clinical experience with SGLT2i in non-diabetic proteinuric CKD, demonstrating that these agents significantly reduce albuminuria while providing cardiovascular and renal benefits comparable to those observed in diabetic CKD [15, 16]. Despite these advances, a considerable gap of knowledge remains regarding their efficacy and safety in patients with LN, as this population is systematically excluded from the largest and most influential clinical trials. Our group recently published a comprehensive review addressing this issue [17]. To further explore this gap, our multidisciplinary team conducted a pilot study in LN patients on stable immunosuppressive therapy and non-immunosuppressive treatment with RAASi to whom a 10-mg dose of empagliflozin was added. This intervention achieved a 50% reduction in residual proteinuria with minimal changes in eGFR and few side effects [18]. Although preliminary, these data suggest that the potential benefits of SGLT2i and other nephroprotective agents may extend to the LN population. In addition, other novel agents with the potential to reduce albuminuria and confer cardiorenal benefits include non-steroid MRAs, glucagon-like peptide-1 receptor agonists, and endothelin receptor antagonists. Nevertheless, the absence of LN-specific evidence prevents firm recommendations, emphasizing the urgent need for translational research that bridges nephrology and autoimmunity.

Our study did not reveal significant differences in short-term cardiovascular or mortality outcomes between patients achieving CRR with normal albuminuria and those with persistent pathological albuminuria. This finding contrasts with previous reports from various European cohorts, which have documented mortality rates in patients with SLE ranging from 13.8 to 16 deaths per 1000 patient-years, and standardized mortality ratios 1.7 to 3.1-fold higher than those observed in the general population. Notably, CVD has emerged as a leading cause of death in these cohorts, accounting for 27–52% of fatalities [17, 19].

Several factors may contribute to this outcome, including the relatively young age of our cohort and the limited duration of follow-up time. Given the well-established association between albuminuria and adverse cardiorenal outcomes, an extended longitudinal observation period may be necessary to accurately assess the long-term cardiovascular impact of persistent albuminuria in this population.

This study has several limitations that should be acknowledged. First, there is a potential selection bias, as the cohort was limited to patients followed in the dedicated LN clinic, excluding those managed in other settings such as advanced CKD or renal transplant clinics, who are likely to present with greater comorbidity and more complex clinical profiles. This may have led to the underrepresentation of patients with more advanced renal damage or comorbidities. Second, the relatively short follow-up period and the young age of the study population limit the ability to detect long-term outcomes, including renal decline, LN flares or cardiovascular events. Consequently, while we report a high prevalence of persistent albuminuria in patients achieving CRR, its direct association with adverse clinical outcomes could not be demonstrated in this cohort, and its clinical significance must be inferred from external literature and pathophysiological considerations. Finally, although multivariable analyses were performed, no statistically significant independent predictors for persistent pathological albuminuria were identified, underscoring the limitations of this cohort in establishing causal associations.

Nonetheless, this study has several strengths, including its real-world clinical setting, the homogeneous follow-up and management protocols applied at a specialized centre, and, to our knowledge, the first work specifically addressing persistent pathological albuminuria in patients achieving CRR within a LN cohort.

## Conclusion

While achieving remission of LN based on conventional criteria represents an important milestone, it may not be sufficient to fully address the underlying kidney dysfunction or to mitigate the risk of long-term cardiovascular events. A substantial proportion of patients who meet the criteria for complete remission may still exhibit persistent pathological albuminuria and likely signifies ongoing subclinical renal injury with long-term cardiovascular and renal implications. This observation demands heightened clinical awareness and proactive intervention, ideally incorporating emerging nephroprotective strategies to optimize both cardiovascular and renal outcomes. These insights could ultimately lead to the consideration of albuminuria not only as a diagnostic and prognostic marker, but also as a modifiable therapeutic target, helping to refine renal remission criteria that more accurately reflect the long-term prognosis of LN.

## Supplementary Material

rkag032_Supplementary_Data

## Data Availability

The data underlying this article cannot be shared publicly due to patient privacy and institutional restrictions. The data will be shared on reasonable request to the corresponding author.
