# Supplementary material for: Albuminuria in Lupus Nephritis: the hidden threat to cardiovascular health
Source: Rheumatol Adv Pract. 2026 Mar 16;10(2):rkag032. doi: 10.1093/rap/rkag032 (PMC13050262; doi:10.1093/rap/rkag032)
Supplement: rkag032_Supplementary_Data [file rkag032_supplementary_data.docx]

**Supplementary tables**

**Supplementary Table S1. Baseline characteristics at lupus nephritis onset among patients included in the overall cohort.**

| Clinical characteristics | |
| --- | --- |
| Patients, N | **91** |
| Female/Male, N (%) | 82/9 (90.1/9.9) |
| Race/Ethnicity, N (%)   - Caucasian - Hispanic - Asian - African | 43 (47.3)  35 (38.5)  8 (8.8)  5 (5.5) |
| Age, years (M ± SD) | 33.19 ± 13.83 |
| Time of follow-up, months (median, IQR) | 78 (38 – 195) |
| Concomitant APS or APA, N (%) | 25 (27.5) |
| HBP, N (%)   - Systolic blood pressure, mmHg (M ± SD) - Diastolic blood pressure, mmHg (M ± SD) | 42 (46.2)  130.12 ± 16.23  81.56 ± 10.79 |
| T2DM, N (%) | 2 (2.2) |
| DL, N (%) | 41 (45.1) |
| Active smoker, N (%) | 11 (12.1) |
| Histological characteristics | |
| ISN/RPS histological classification, N (%)   - Class II - Class III - Class IV - Class V - Class III + V - Class IV + V | 3 (3.3)  15 (16.5)  34 (37.4)  16 (17.6)  8 (8.8)  15 (16.5) |
| Activity Index (median, IQR) | 5 (0 – 9) |
| Chronicity Index (median, IQR) | 1 (0 – 2) |
| Biochemical characteristics | |
| sCr titer, mg/dl, (M ± SD) | 0.96 ± 0.57 |
| eGFR ml/min, (M ± SD) | 81.83 ± 15.30 |
| uPCR, g/g (median, IQR) | 1.82 (0.80 – 4.96) |
| uACR, mg/g (median, IQR) | 994.45 (506.88 – 3055.73) |
| Fasting blood glucose (M ± SD) | 88.85 ± 20.63 |
| Total cholesterol titer, mg/dL (M ± SD) | 184.55 ± 36.02 |
| LDL-cholesterol titer, mg/dL (M ± SD) | 101.96 ± 28.49 |
| Triglycerides titer, mg/dL (M ± SD) | 98.02 ± 39.97 |
| Immunological features | |
| C3 titer, mg/dL (median, IQR) | 59.90 (43.60 – 84.30) |
| C4 titer, mg/dL (median, IQR) | 11.10 (5.40 – 18.25) |
| ANA status positive, N (%) | 51 (56) |
| Anti-dsDNA status positive, N (%) | 37 (40.7) |
| SLE-DAI 2K (median, IQR) | 14 (11 – 20) |
| SLE-DAS (median, IQR) | 20.10 (14.66 – 32.81) |

*Abbreviations: APS (anti-phospholipid Syndrome),* *APA (anti-phospholipid antibodies), HBP (high blood pressure), T2DM (type 2 diabetes mellitus), DL (dyslipidemia), ISN/RPS (International Society of Nephrology/Renal Pathology Society), sCr (serum creatinine), eGFR (estimated glomerular filtration rate), uPCR (urinary protein-creatinine ratio); uACR (urinary albumin-creatinine ratio);* *ANA (antinuclear antibodies); Anti-dsDNA (anti-double-stranded DNA); SLE-DAI 2K (Systemic Lupus Erythematosus Disease Activity Index 2000); SLE-DAS (Systemic Lupus Erythematosus Disease Activity Score); M ± SD (mean ± standard deviation); IQR (interquartile range)*

**Supplementary Table S2. Treatment employed at the end of follow-up among patients meeting criteria for CRR.**

| Patients, N | 77 |
| --- | --- |
| Hydroxychloroquine, N (%) | 60 (70.9) |
| Statins, N (%) | 34 (44.2) |
| Acetylsalicylic acid, N (%) | 24 (31.2) |
| Patients undergoing antiproteinuric treatment, N (%) | 61 (79.2) |
| Number of antiproteinuric agents, median (IQR)   - 1 antiproteinuric, N (%) - 2 antiproteinurics, N (%) - 3 antiproteinurics, N (%) - 4 antiproteinurics, N (%) | 1 (1 – 2)  34 (44.2)  15 (19.5)  11 (14.3)  1 (1.3) |
| Class of antiproteinuric agent administered, N (%)   - ACEi/ARBs - SGLT2i - Thiazides - MRAs | 60 (77.9)  14 (18.2)  10 (13)  15 (19.5) |
| Type of immunosuppression, N (%)   - GC - MMF - CNi - Belimumab - GC + MMF - GC + CNi - GC + Belimumab - GC + Metotrexate - MMF + CNi - MMF + Belimumab - GC + MMF + CNi - GC + MMF + Belimumab - GC + MMF + CNi + Belimumab - Others | 5 (6.5)  19 (24.7)  2 (2.6)  4 (5.2)  9 (11.7)  2 (2.6)  1 (1.3)  1 (1.3)  1 (1.3)  4 (5.2)  3 (3.9)  3 (3.9)  5 (6.5)  2 (2.6) |

*Abbreviations:* *ACEi/ARBs (angiotensin-converting enzyme inhibitors / angiotensin II receptor blockers), SGLT2i (sodium-glucose cotransporter-2 inhibitors), MRAs (mineralocorticoid receptor antagonists), GC (glucocorticoids), MMF (mycophenolate mofetil), CNi (calcineurin inhibitors)*

**Supplementary Table S3. Clinical, immunological and biochemical characteristics of patients meeting criteria for CRR at the end of follow-up.**

| Patients, N | 77 | |
| --- | --- | --- |
| Female/Male, N (%) | 68/9 (88.3/11.7) | |
| Age, years (M ± SD) | 45.71 ± 14.06 |  |
| LN recurrence, N (%) | 12 (15.6) |  |
| Concurrent APS, N (%) | 23 (29.9) |  |
| Ischemic cardiac disease, N (%) | 0 (0) | |
| Major cardiovascular event, N (%) | 1 (1.3) | |
| SCr titer, mg/dl (M ± SD) | 0.89 ± 0.44 | |
| eGFR, ml/min (M ± SD) | 77.48 ± 19.68 | |
| uPCR, g/g (median, IQR) | 0.16 (0.10 – 0.28) | |
| uACR, mg/g (median, IQR) | 43.90 (11.28 – 99.47) | |
| ANA status positive N (%) | 12 (15.6) | |
| Anti-dsDNA status positive, N (%) | 8 (10.4) | |
| SLE-DAI 2K, median (IQR) | 0 (0 – 2) | |
| SLE-DAS, median (IQR) | 0.37 (0.37 – 1.12) | |
| BMI, kg/m² (median, IQR) | 24.40 (21.50 – 28.70) | |
| Low C3, N (%) | 40 (51.9) | |
| Low C4, N (%) | 27 (35.1) | |
| C3 titer, mg/dL (M ± SD) | 82.10 (71.70 – 90) | |
| C4 titer, mg/dL (M ± SD) | 16.80 (12.90 – 23.65) | |

*Abbreviations: LN (lupus nephritis), Cr (creatinine), eGFR (estimated filtrate glomerular rate),* *ANA (antinuclear antibodies), Anti-dsDNA (anti-double-stranded DNA), SLE-DAI 2K (Systemic Lupus Erythematosus Disease Activity Index 2000), SLE-DAS (Systemic Lupus Erythematosus Disease Activity Score), BMI (body mass index).*
